# Meta-analysis of quantitative diffusion-weighted MR imaging in the differential diagnosis of breast lesions

**DOI:** 10.1186/1471-2407-10-693

**Published:** 2010-12-29

**Authors:** Xin Chen, Wen-ling Li, Yi-li Zhang, Qian Wu, You-min Guo, Zhi-lan Bai

**Affiliations:** 1 Department of Radiology, Second Affiliated Hospital of Medical College of Xi'an Jiaotong University, Xi'an, Shannxi, China; 2 Department of Radiology, First Affiliated Hospital of Medical College of Xi'an Jiaotong University, Xi'an, Shannxi, China; 3 Department of Epidemiology, Medical College of Xi'an Jiaotong University, Xi'an, Shannxi, China

## Abstract

**Background:**

To determine, in a meta-analysis, the diagnostic performance of quantitative diffusion-weighted (DW) MR imaging in patients with breast lesions.

**Methods:**

English and Chinese studies published prior to June 2009 to assess the diagnostic performance of quantitative DWI in patients with breast lesions were reviewed and summarized with reference to the inclusion and exclusion criteria. Methodological quality was assessed by using the quality assessment of diagnostic studies (QUADAS) instrument. Publication bias analysis was performed by using Comprehensive Meta-analysis version 2. Meta-Disc version 1.4 was used to describe primary results and explore homogeneity by Chi-square test and inconsistency index; to explore threshold effect by receiver operator characteristic (ROC) space and Spearman correlation coefficient; and to pool weighted sensitivity and specificity by fixed or random effect model. A summary ROC (sROC) curve was constructed to calculate the area under the curve (AUC).

**Results:**

Of 65 eligible studies, 13 with 615 malignant and 349 benign lesions were included in the original meta-analysis, among which heterogeneity arising from factors other than threshold effect and publication bias was explored. Methodological quality was moderate. The pooled weighted sensitivity and specificity with corresponding 95% confidence interval (CI) in one homogenous subgroup of studies using maximum b = 1000 s/mm^2 ^were 0.84 (0.80, 0.87) and 0.84 (0.79, 0.88) respectively. AUC of sROC was 0.9085. Sensitivity analysis demonstrated that the pooled estimates were stable and reliable.

**Conclusions:**

Quantitative DWI has a higher specificity to differentiate between benign and malignant breast lesions compared to that of contrast-enhanced MRI. However, large scale randomized control trials (RCTs) are necessary to assess its clinical value because of disunified diffusion gradient factor b and diagnosis threshold.

## Background

Breast cancer is the most common cancer in women worldwide [1]. Despite the improvement in the detection of breast cancer with the widespread application of mammography and ultrasound, differentiation between benign and malignant breast lesions remains a difficult diagnosis problem, especially in dense fibroglandular breasts. Numerous studies have been performed to assess the diagnostic performance of contrast-enhanced magnetic resonance imaging (MRI) in breast lesions [2-5]. Based on the enhancement pattern of lesions and morphologic changes, breast MRI offers an overall sensitivity of 90% and specificity of 72% in detecting breast lesions according to a published Meta-analysis [6]. Therefore, the classification of a lesion detected on MRI as benign or malignant remains a challenge [6,7].

One of the latest advancements in MRI technology is the application of diffusion-weighted imaging (DWI) to offer quantitative evaluation of apparent diffusion coefficient (ADC). Quantitative DWI is a powerful imaging tool which provides unique information related to the diffusion of water molecules in the tissue and allows estimation of cellularity and tissue structure [8]. Restricted water movement in tumors with high cellularity usually leads to higher signal intensity and smaller ADC value [7,9]. ADC is measured by acquiring the MR signal at least twice, typically with and without diffusion weighting by the following formula: ADC = [In (S_0_/S_b_)]/b, where S_b _and S_0 _are the signal intensities on the DW imaging with and without diffusion weighting, respectively [10]. Theoretically, the diffusion sensitivity is easily varied by changing the diffusion gradient factor known as the "b value" which is proportional to the gradient amplitude, the duration of the applied gradient and the time interval between the paired gradients [8].

The potential role of quantitative DWI in characterizing breast lesions has been reported [11-18]. However, the reported sensitivity and specificity of diagnosing malignant breast tumors ranged from 62.5% to 92.8% and 45.8% to 96.7%, respectively. Comparison of the diagnostic performance of breast quantitative DWI among the studies may have been compromised by differences in the patient characteristics, MR imaging techniques, and diagnostic criteria for malignancy in the studies. In this study, we pool a number of DWI studies of the breast to evaluate the diagnostic performance of DWI in breast lesion characterization.

## Methods

### Literature Search

The MedLine and CNKI (China National Knowledge Infrastructure) search were performed (Li WL and Bai ZL; MAY 20, 2009) by using the terms "Diffusion-Weighted Imaging [MeSH] or DWI" for the diagnostic test and "Breast Neoplasms [MeSH] or breast cancer or breast lesions or breast" for the clinical domain. Terms regarding diagnostic performance were excluded in our search criteria to minimize missing relevant studies. We limited our search to publications in English and Chinese languages, female subjects, search term presence in the title or abstract of the article and publication date no later than May 2009. The Cochrane library, Ovid, Elsevier and Springer databases were also searched (Li WL; June 10, 2009) for research articles with the same criteria. Review articles, letters, comments, case reports and unpublished articles were excluded. Extensive cross-checking of the reference lists of all retrieved articles was performed to supplement the list of articles.

### Selection of Articles

Inclusion criteria were: varied pathology within the dataset; total number of lesions ≥ 30, with the number of both malignant and benign lesions each ≥ 10; histopathologic analysis (performed at surgery and biopsy) and follow-up by ultrasound, mammography, or MRI used as the reference standards; Sufficient information were presented to calculate the true-positive (TP), false-positive (FP), true-negative (TN) and false-negative (FN) values for per-lesion statistics; When same data or subsets of data were presented in more than one article, the article with the most details and/or most recently published was chosen.

Articles were selected by two steps according to the road map of diagnostic systematic reviews and guidelines [19]. First, articles were excluded after inclusion and exclusion criteria were applied to the titles and abstracts of the articles fulfilling the search criteria. We then determined the final studies included in the meta-analysis after applying the same inclusion and exclusion criteria to the remaining content of the articles.

Studies were excluded if results of different MR imaging series (such as contrast-enhanced imaging and DWI) were presented in combination and could not be differentiated for performance assessment of tests on only DWI series.

### Quality Assessment and Data Extraction

Two observers (Chen X and Zhang YL) independently selected eligible articles and extracted relevant data about study characteristics and examination results from each article by using a standardized form. To resolve disagreement between reviewers, a third reviewer (Guo YM) assessed all involved items. The majority opinion was used for analysis.

Methodological quality of included studies was assessed independently by two observers using the quality assessment of diagnostic studies (QUADAS) instrument, a quality assessment tool specifically developed for systematic reviews of diagnostic accuracy studies [20,21].

The author, study nation, year of publication, number and age of subjects, and diagnostic test characteristics including b values, mean ADC values of malignant and benign lesions, and diagnostic threshold for malignant lesions were extracted from each study.

Values for TP, FP, TN, FN, sensitivity (SEN), specificity (SPE), accuracy (ACC), positive predictive value (PPV), negative predictive value (NPV), positive likelihood ratio (PLR) and negative likelihood ratio (NLR) results in the detection of malignant lesions were extracted. SEN, SPE, ACC, PPV, NPV, PLR and NLR were calculated by the following formulas:

$$\begin{array}{l} \text{SEN}=\text{TP}/\left(\text{TP}+\text{FN}\right)\\ \text{SPE}=\text{TN}/\left(\text{FP}+\text{TN}\right)\\ \text{ACC}=\left(\text{TP}+\text{TN}\right)/\left(\text{TP}+\text{FP}+\text{TN}+\text{FN}\right)\\ \text{PPV}=\text{TP}/\left(\text{TP}+\text{FP}\right)\\ \text{NPV}=\text{TN}/\left(\text{FN}+\text{TN}\right)\\ \text{PLR}=\text{SEN}/\left(\text{1}-\text{SPE}\right)\\ \text{NLR}=\left(\text{1}-\text{SEN}\right)/\text{SPE} \end{array}$$

All tabulated results for different readers (interobserver), for multiple observations per reader (intraobserver) and for multiple b values or techniques were counted as separate data sets.

### Meta-Analysis

#### Homogeneity test

We used the *Q *statistic of Chi-square value test and inconsistency index (I-squared, *I^2^*) to estimate the heterogeneity of individual studies contributing to the pooled estimate. The homogeneity was to evaluate if the differences across the studies were greater than expected by chance alone. *P *< 0.05 suggests presence of heterogeneity beyond what could be expected by chance alone. I-squared (*I^2^*) describes the percentage of total variation across studies due to heterogeneity rather than chance and was also used as a measure to quantify the amount of heterogeneity. *I*^2 ^> 50% suggests heterogeneity [22].

#### Threshold effect

Different thresholds may be used in included studies to define a positive test result due to lack of standardization. Differential threshold effect may be the reason for detectable difference in sensitivities and specificities of test accuracy studies. Representation of accuracy estimates from each study in a receiver operating characteristic (ROC) space and computation of Spearman correlation coefficient between the log (SEN) and log (1-SPE) were assessed for threshold effect. A typical pattern of "shoulder arm" plot in a ROC space and a strong positive correlation would suggest threshold effect [23,24].

#### Publication bias analysis

Studies with optimistic results are more likely to be submitted and accepted for publication than studies with unfavorable results. Since publication biases would tend to exaggerate clinical effects resulting in potentially erroneous clinical decision making, it is important to assess the likely extent of the bias and its potential impact on the conclusions [25]. Publication bias can be visually examined after construction of a funnel plot and quantitatively detected by fail-safe N (NFS). In the absence of publication bias, the data points form a symmetric funnel-shaped distribution, whereas an asymmetric distribution indicates the presence of publication bias [26]. NFS refers to the exact number of hypothetical studies with null results that would be required to nullify the statistical significance of combined effect [27]. A relatively small NFS should be cause for concern. However, if NFS is large, we can be confident the combined effect, while possibly inflated by the exclusion of some studies, is nevertheless not zero [28].

#### Statistical pooling

Statistical pooling is not always necessary in every systematic review of test accuracy studies. The necessary precondition for pooling estimates is that the studies and results are reasonably homogeneous. The estimates can be pooled by the fixed effect model (FEM) or by the random effect model (REM) to incorporate variation among studies, and the output can be presented graphically as forest plots. If heterogeneity due to threshold effect is present, the accuracy data can be pooled by fitting a summary ROC (sROC) curve and summarizing that curve by means of the area under the curve (AUC). A sROC curve summarizes and combines the true and false positive rates from different diagnostic studies. The overall performance of diagnostic studies can be visualized and reflected by a sROC curve without being affected by a change of threshold values [29]. The best diagnostic modality would yield a point in the upper left corner or coordinate (0, 1) of the sROC space, representing 100% sensitivity (no false negatives) and 100% specificity (no false positives) at the individual subject level. Similarly, AUC ranges from 1 for a perfect test that always diagnoses correctly, to 0 for a test that never does so in single studies or meta-analyses. If there is heterogeneity due to sources other than threshold effect, pooling should only be attempted within homogeneous subsets [23].

#### Sensitivity analysis

The pooled estimates were reappraised when suspicious studies were excluded, and the reappraised results were compared with the original results to assess stability and reliability of our meta-analysis.

The homogeneity test, threshold effect analysis, pooled weighted sensitivity and specificity, sROC curve and sensitivity analysis were performed by using Meta-Disc version 1.4 [23]. Publication bias analysis was performed by using Comprehensive Meta-analysis version 2 [30].

## Results

With the computer search and manual cross-checking of reference lists, 65 abstracts (26 English and 39 Chinese) were retrieved. After reading the titles and abstracts, we found 25 eligible articles (15 English and 10 Chinese). After reading the full texts, we excluded 12 of the 25 relevant articles for the following reasons: The objective of studies was not to explore the diagnostic performance of 1.5 T MR DWI on breast lesions [31-34] (*n *= *4*); Benign breast lesions in the study were all fibroadenomas [35] (*n = 1*); Researchers did not report data that could be used to construct or calculate TP, FP, TN and FN results [15,17,36] (*n *= 3); Researchers presented results from a combination of different MR series that could not be differentiated for assessment of single DWI [37] (*n = 1*); Data or subsets of data were presented in other articles [38,39] (*n = 2*). Thirteen articles (5 English and 8 Chinese) fulfilled all inclusion and exclusion criteria and were selected for data extraction and data analysis [11,13,14,16,18,40-47].

Nine hundred and sixty four breast lesions (615 malignant, 349 benign) were included from 13 studies. The mean ADC values of malignancy ranged from 0.87 to 1.36 × 10^-3 ^mm^2^/s. The mean ADC values of benign lesions ranged from 1.00 to 1.82 × 10^-3 ^mm^2^/s. The cutoff values differentiating malignant and benign lesions ranged from 0.90 to 1.76 × 10^-3 ^mm^2^/s while the sensitivity and specificity ranged from 63% to 100% and 46% to 97%, respectively. The abstracted data of these individual studies are summarized in Tables 1 and 2. Two or multiple subsets of data in one study were included because: The two methods of echo planar imaging (EPI) and half-Fourier acquired single-shot turbo spin echo (HASTE) were used to perform breast DWI in Baltzer et al. [40]; Marini et al. used 1.1 × 10^-3 ^mm^2^/s (mean diffusivity of benign lesions - 1 SD) and 1.31 × 10^-3 ^mm^2^/s (mean diffusivity of malignant lesions -2 SD) as cutoff between benign and malignant breast lesions [18]; ADC values were measured by two radiologists independently in Rubesova et al. [14]; Multiple b values were used in some studies [41,42,44,45].

**Table 1:** The characteristics of included studies.

Author	Publication Year	Country	Mean (range) age (years)	No. of patients	No. of total lesions	No. of benign lesions	No. of malignant lesions
Baltzer(40)	2009	Germany	54 (N)	65	74	35	39
Marini(18)	2007	Italy	53 (24-79)	60	63	21	42
Rubesova(14)	2006	Belgium	52 (25-74)	78	87	22	65
Woodhams(13)	2005	Japan	53 (14-88)	190	191	24	167
Guo(11)	2002	China	58 (25-75)	52	55	24	31
Huang(41)	2008	China	50 (31-77)	N	56	24	32
Jin(42)	2008	China	N (N)	56	60	20	40
Tang(43)	2008	China	51 (33-76)	48	70	25	45
Gu(44)	2008	China	51 (33-83)	83	95	52	43
Lou(45)	2007	China	42 (18-71)	50	58	26	32
Luo(16)	2007	China	43 (24-65)	52	60	33	27
Li(46)	2005	China	45 (24-68)	35	41	13	28
Zhao(47)	2005	China	43 (21-72)	46	54	30	24

Note: N = not mentioned

**Table 2:** The ADC measurement of included studies (×10-3mm2/s) ($\bar{\text{x}}$ ± SD).

Author	B value (s/mm^2^)	Mean ADC of malignant	Mean ADC of benign	Mean ADC of normal	Threshold
Baltzer	0, 750, 1000	1.05 ± 0.33	1.63 ± 0.42	N	1.23
	0, 800	1.09 ± 0.38	1.67 ± 0.40	N	1.24
Marini	0, 1000	0.95 ± 0.18	1.48 ± 0.37	N	1.10
					1.31
Rubesova	0, 200, 400, 600, 800, 1000	0.95 ± 0.02	1.51 ± 0.07	N	1.15
		0.99 ± 0.03	1.47 ± 0.08	N	1.10
Woodhams	0, 750	1.22 ± 0.31	1.67 ± 0.54	2.09 ± 0.27	1.60
Guo	0, 1000	0.97 ± 0.20	1.57 ± 0.23	N	1.30
Huang	0, 500	1.02 ± 0.18	1.61 ± 0.32	1.67 ± 0.21	1.32
	0, 1000	0.99 ± 0.16	1.59 ± 0.33	1.65 ± 0.21	1.25
Jin	0, 600	1.33 ± 0.36	1.82 ± 0.31	2.05 ± 0.33	1.44
	0, 1000	1.08 ± 0.32	1.61 ± 0.33	1.85 ± 0.33	1.18
Tang	0, 800	1.15 ± 0.19	1.47 ± 0.25	N	N
	0, 1000	1.08 ± 0.19	1.42 ± 0.26	N	1.38
Gu	0, 500	1.36 ± 0.38	1.64 ± 0.34	1.77 ± 0.39	1.5
	0, 1000	1.18 ± 0.31	1.39 ± 0.32	1.56 ± 0.33	1.3
	0, 2000	0.82 ± 0.20	1.00 ± 0.23	0.90 ± 0.27	0.90
Lou	0, 400	1.28 ± 0.48	1.71 ± 0.42	2.06 ± 0.48	1.76
	0, 600	1.18 ± 0.41	1.59 ± 0.41	1.92 ± 0.53	1.64
	0, 800	1.11 ± 0.41	1.70 ± 0.34	1.82 ± 0.48	1.52
	0, 1000	1.05 ± 0.38	1.55 ± 0.35	1.75 ± 0.52	1.43
Luo	0, 800	0.87 ± 0.23	1.59 ± 0.26	1.98 ± 0.31	1.22
Li	0, 1000	1.21 ± 0.26	1.49 ± 0.43	N	1.42
Zhao	0, 1000	0.91 ± 0.25	1.58 ± 0.22	1.78 ± 0.51	1.01

Note: N = not mentioned; ADC = apparent diffusion coefficient; SD = standard deviation.

Results of distribution of study design characteristics in 13 studies according to QUADAS items are shown in Table 3. Most studies have a suboptimal design in regard to the reporting of selection criteria (67.2% for "yes" responses to question 2), the description of the execution of the reference standard (76.9% for "no" responses to question 9), the interpretation of the reference standard results without knowledge of the index test results and the interpretation of the index test results without knowledge of the reference standard (84.6% and 100% for "unclear" responses to question 10 and 11 respectively), reporting of uninterpretable and/or intermediate test results (69.2% for "no" responses to question 13) and explanation of withdrawals from the study (76.9% for "no" responses to question 14).

**Table 3:** Evaluation of quality of included studies using the QUADAS tool.

Question about study design characteristic														Total		
	
	Baltzer	Marini	Rubesova	Woodhams	Guo	Huang	Jin	Tang	Gu	Lou	Luo	Li	Zhao	Yes (%)	No (%)	Unclear (%)
1 Patient spectrum	Y	Y	Y	Y	Y	Y	Y	Y	Y	Y	Y	Y	Y	100	0	0
2 Reporting of selection critetia	Y	Y	Y	Y	N	N	Y	Y	N	N	Y	Y	Y	69.2	30.8	0
3 Reference standard	Y	Y	Y	Y	Y	Y	Y	Y	Y	Y	Y	Y	Y	100	0	0
4 Absence of disease progression bias	Y	Y	Y	Y	Y	Y	Y	Y	Y	Y	Y	Y	Y	100	0	0
5 Absence of partial vertification bias	Y	Y	Y	Y	Y	Y	Y	Y	Y	Y	Y	Y	Y	100	0	0
6 Absence of differential vertification bias	N	N	Y	Y	N	Y	Y	Y	Y	Y	Y	N	Y	69.2	30.8	0
7 Absence of incorporation bias	Y	Y	Y	Y	Y	Y	Y	Y	Y	Y	Y	Y	Y	100	0	0
8 Description of index text execution	Y	Y	Y	Y	Y	Y	Y	Y	Y	Y	Y	Y	Y	100	0	0
9 Description of reference standard execution	Y	N	N	N	Y	N	N	N	Y	N	N	N	N	23.1	76.9	0
10 Absence of test review bias	Y	U	U	U	U	Y	U	U	U	U	U	U	U	15.4	0	84.6
11 Absence of diagostic review bias	U	U	U	U	U	U	U	U	U	U	U	U	U	0	0	100
12 Absence of clinical review bias	Y	Y	Y	Y	Y	Y	Y	Y	Y	Y	Y	Y	Y	100	0	0
13 Reporting of uninterpretable/intermediate results	N	N	Y	N	Y	N	N	Y	N	N	Y	N	N	30.8	69.2	0
14 Withdrawal	Y	U	Y	U	Y	U	U	U	U	U	U	U	U	23.1	76.9	0

Note: Y = yes; N = no; U = unclear; QUADAS = quality assessment of diagnostic studies.

**Figure 1:**
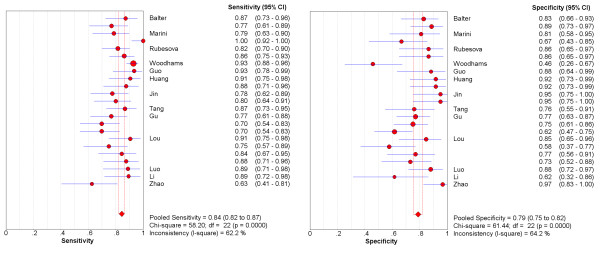
**Forest plots of sensitivity and specificity, with corresponding 95% CIs from all eligible studies**.

**Figure 2:**
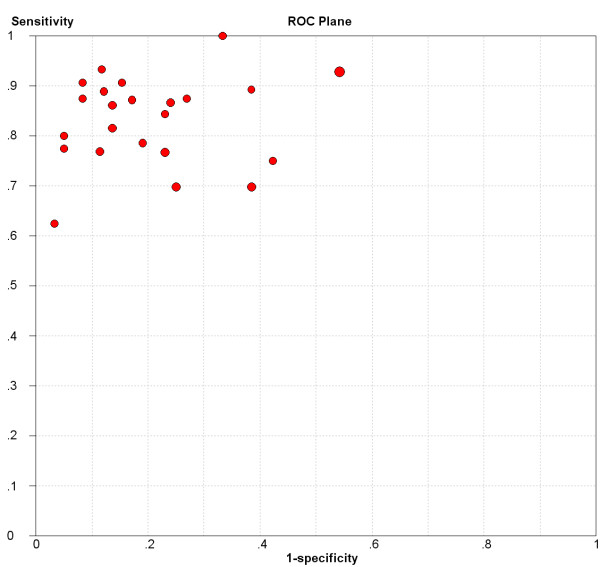
**Sensitivity and 1-specficity plotted in receiver operating characteristic space for individual studies**.

The forest plots of sensitivities and specificities from 23 subsets of data from all 13 studies are shown in Figure 1. A homogeneity test of sensitivity and specificity shows *Q *= 58.20 (*P *< 0.0001), *I*^2 ^= 62.2% and *Q *= 61.44 (*P *< 0.0001), *I*^2 ^= 64.2%, respectively. Therefore, notable heterogeneities are detected. The next step is the representation of sensitivity against 1-specificity from each study in a ROC space to explore the threshold effect. The pattern of the points in this plot is not a "shoulder-arm" shape (Figure 2). A Spearman rank correlation is performed as a further test for threshold effect. The Spearman correlation coefficient was equal to 0.097 (*P *= 0.66) and indicates that there should be other factors than threshold effect resulting in variations in accuracy estimates among individual studies.

**Figure 3:**
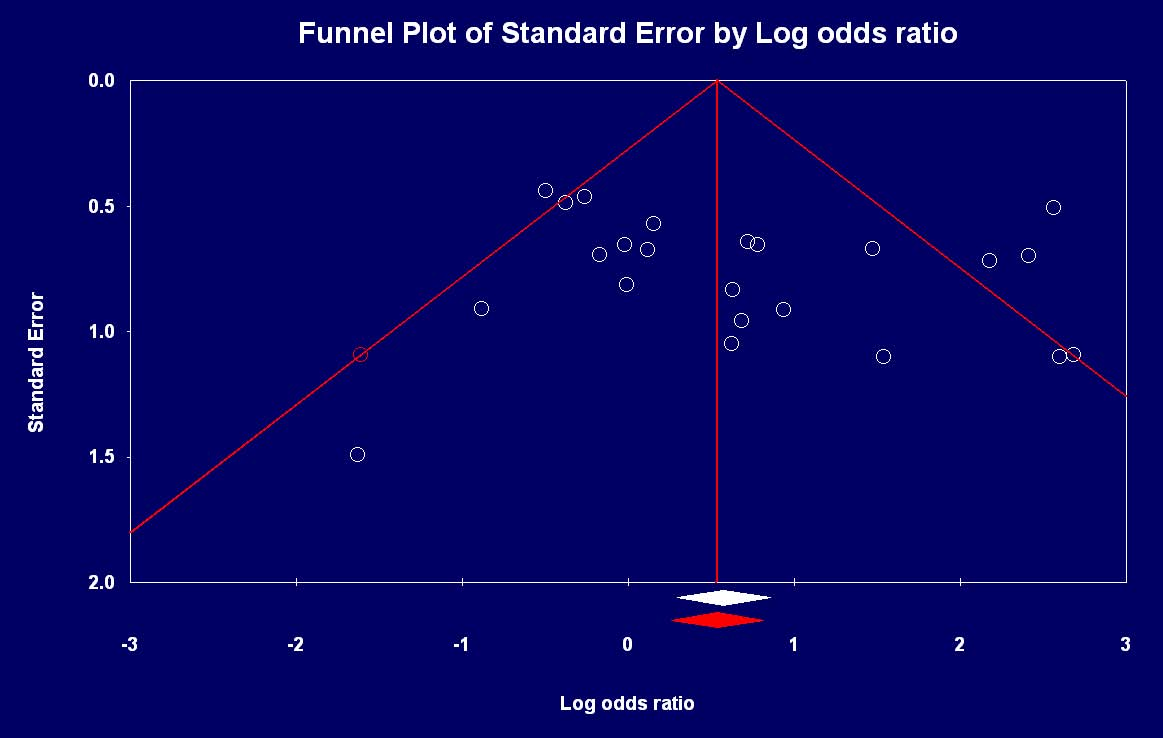
**Funnel plot of eligible studies**. Note: White flakes represent published articles, and red flake represents possibly missed article. White and red rhombuses represent actual and theoretical combined effect size respectively.

The funnel plot in Figure 3 shows that the studies are distributed symmetrically about the combined effect size and yield a z-value of 4.46191 with corresponding *P*-value of 0.00001. The NFS is 92. This means that we would need to locate and include 92 "null" studies in order for the *P *to exceed 0.05. There is no significant publication bias, and actual combined effect size is equal to the theoretical combined effect size.

**Figure 4:**
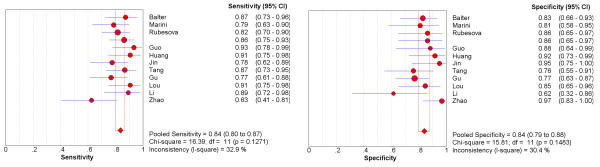
**Forest plots of sensitivity and specificity, with corresponding 95% CIs from studies using maximum b = 1000 s/mm^2^**.

**Figure 5:**
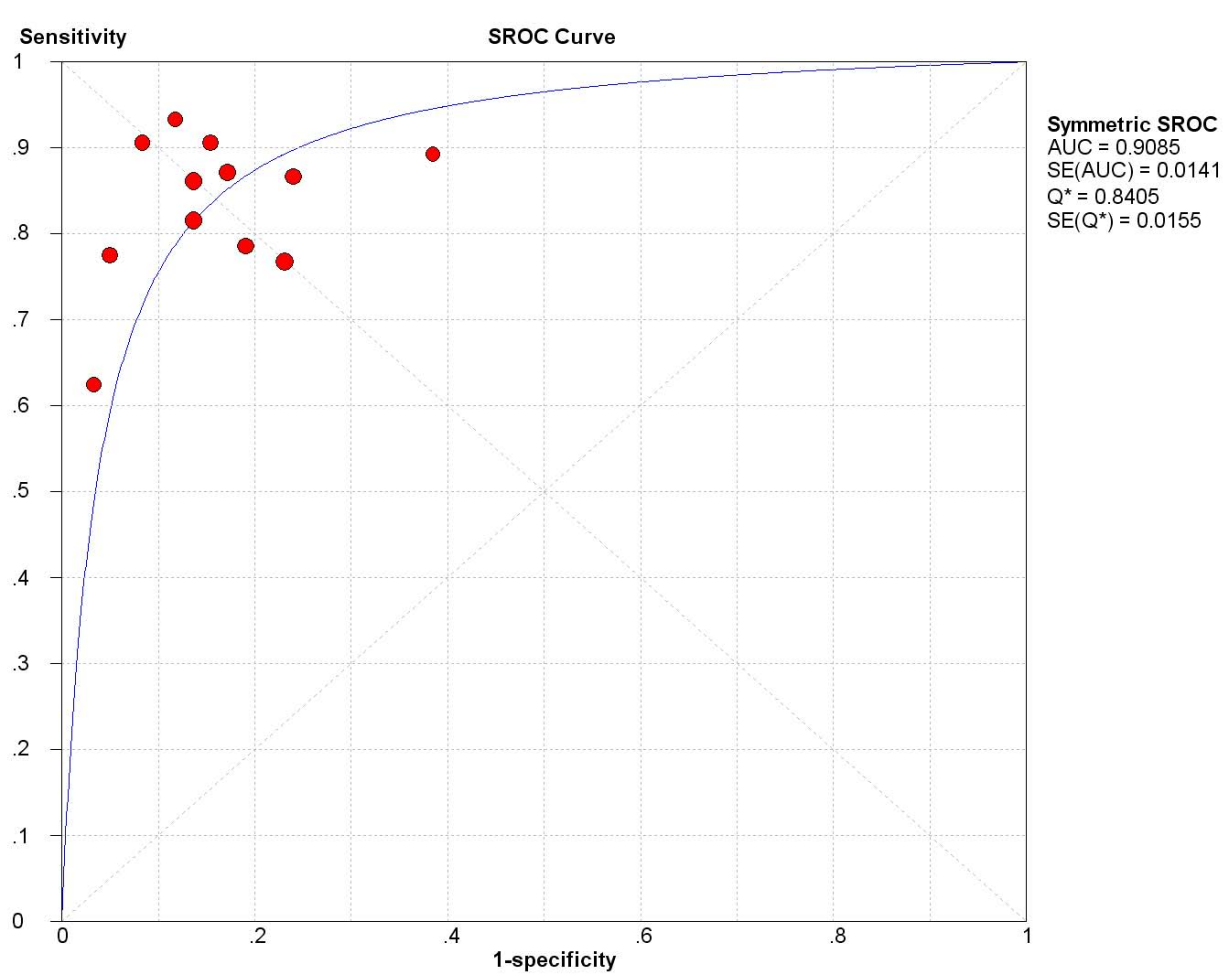
**SROC curve for studies using maximum b = 1000 s/mm^2^**.

Having found notable heterogeneity beyond threshold effect and publication bias, we then focus on the subgroup of studies using maximum b = 1000 s/mm^2^. A homogeneity test of sensitivity and specificity shows *Q *= 16.39 (*P *= 0.1271), *I*^2 ^= 32.9% and *Q *= 15.81 (*P *= 0.1483), *I*^2 ^= 30.4% respectively. The pooled weighted sensitivity and specificity with corresponding 95% CIs are 0.84 (0.80, 0.87) and 0.84 (0.79, 0.88) (Figure 4) respectively, and expressed as AUC of sROC curve is 0.9085 (Figure 5).

Heterogeneity, the pooled weighted sensitivity and specificity, and AUC are analyzed again when [46] and [47] were excluded respectively due to the seemingly differential sensitivity and specificity from others (Table 4). The pooled sensitivity and specificity is similar, and the corresponding 95% CIs are predominantly overlapped with each other. The homogeneity is not reversed. The results of sensitivity analysis demonstrate that the pooled estimates are stable and reliable.

**Table 4:** Results of heterogeneity and the pooled estimations excluding suspicious studies.

Study	Sensitivity (95%CI)	Heterogeneity		Specificity (95% CI)	Heterogeneity		AUC
					
		*P*	*I^2^*		*P*	*I^2^*	
b = 1000	0.84(0.80, 0.87)	0.1271	32.9%	0.84(0.79, 0.88)	0.1483	30.4%	0.9085
Excluding Li's	0.83(0.79, 0.86)	0.1153	35.4%	0.85(0.80, 0.89)	0.2999	15.1%	0.9117
Excluding Zhao's	0.85(0.80, 0.87)	0.4180	2.3%	0.84(0.79, 0.88)	0.6377	0	0.9118

Note: AUC = area under the curve; CI = confidence interval.

## Discussion

In this meta-analysis, we calculate an overall sensitivity of 0.84 (95% CI: 0.82, 0.87) and specificity of 0.79 (95% CI: 0.75, 0.82) from 13 studies fulfilling all inclusion and exclusion criteria. However, there is notable heterogeneity among individual studies. Therefore, it is critical to investigate the source of heterogeneity to determine the potential impact factors and to evaluate the appropriateness of statistical pooling of accuracy estimates from various studies.

Meta-Disc is performed to assess threshold effect from representation of accuracy estimates from each study in a ROC space, and Spearman correlation coefficient is computed between the log (SEN) and log (1-SPE). Lack of "shoulder-arm" shape of the points in the ROC space and mild Spearman correlation coefficient (0.097) indicate that there should be factors other than differences in cutoff points for malignancy causing variations in accuracy estimates across individual studies. Additionally, publication bias is an usual source of heterogeneity in meta-analysis. Publication biases might exaggerate clinical effects resulting in potentially erroneous clinical decision making. In systematic reviews or meta-analysis, a thorough literature search is crucial to identify all relevant studies. In this meta-analysis, the search of several electronic databases is supplemented by checking references of relevant studies in order to reduce publication bias [25]. Although only English and Chinese articles are searched, the funnel plot and NFS analysis indicate that there is no significant publication bias in our meta-analysis.

The parameters used in DWI sequences may affect the results of ADC calculation, and the diffusion gradient factor b is one of important parameters [8]. The variability in b values across studies makes the ranges and thresholds of ADC values difficult to interpret. Because b values used in the included studies varied, we needed to explore whether b values were the source of heterogeneity. Meta-analysis is performed again in the subgroup of studies using maximum b = 1000 s/mm^2 ^because more studies used b at 1000 s/mm^2 ^than any other value from Table 2. The results show that there is no significant heterogeneity, and the pooled sensitivity and specificity with 95% CIs are 0.84 (0.80, 0.87) and 0.84 (0.79, 0.88) respectively. The area under the curve of sROC is 0.9085. Compared with the original results from all 13 eligible studies, the specificity is improved. However, we could not determine effect of other b values to pooled accuracy estimates because of the relatively small number of included studies. Furthermore, sensitivity analysis is performed in the subgroup of b = 1000 s/mm^2^. When the studies with the maximum variance of sensitivity and specificity are excluded in sequence, the characteristics of heterogeneity are not changed and the variances of the pooled accuracy estimates are not significant. The sensitivity analysis shows that this meta-analysis in the subgroup is stable.

A previously published meta-analysis for contrast-enhanced MRI including 44 studies reported the overall sensitivity of 0.90 (95% CI: 0.88, 0.92) and specificity of 0.72 (95% CI: 0.67, 0.77) in patients with breast lesions [6]. In comparison, our meta-analysis indicates that DWI has higher specificity but lower sensitivity. However, several differences and limitations in our meta-analysis should be noted. The significance of Peter et al. [6] lies in the diagnostic performance of MR imaging of small lesions detected at mammographic screening. Because a large proportion of these lesions are nonpalpable, they only include studies that enrolled at least one patient with nonpalpable lesion [6]. In our meta-analysis, we do not filter the studies using such criteria. In the included studies, Marini et al. selected lesions with diameter >1 cm [18]. Rubesova et al. selected lesions with diameter > 0.7 cm [14], and Tang et al. selected lesions with diameter ≤ 2 cm [43]. Lesion diameter ranged from 0.3 to 9 cm for other included studies. It is not certain that smaller lesions could be more easily missed in DW images since no included studies reported the number of nonpalpable lesions. Such variation might affect the overall estimates of the sensitivity and specificity. Because MR is one of the important methods for breast cancer diagnosing, DWI, having the advantages of a short examination time and no need to inject a contrast medium could be used in screening crowd to early detect nonpalpable lesions and to generate significant benefits to early tumor diagnosis and human health care needs in the future.

One limitation of our study is the suboptimal quality of included studies. Meta-analysis combines or integrates the results of several independent studies. The quality and reliability of a meta-analysis depends on the quality of included studies. We use the QUADAS tool for assessing methodological quality of individual studies. This tool was specifically developed for quality assessment of diagnostic accuracy studies included in systematic reviews and has been used to help identify severe methodological shortcomings [21]. Most included studies in this meta-analysis had a suboptimal design in regard to the reporting of selection criteria, the description of the execution of the reference standard, the interpretation of the reference standard results without knowledge of the index test results, the interpretation of the index test results without knowledge of the reference standard, reporting of uninterpretable and/or intermediate test results, or explanation of withdrawals from the study (Table 3).

In test accuracy studies, interpretation of the results of the index test may be influenced by knowledge of the results of the reference standard, and vice versa. This is known as review bias and may lead to inflated measures of diagnostic accuracy. As the index test, DWI was always performed first, and interpretation of the results of the DWI was usually done without knowledge of the results of the reference standard. However, if test result was interpreted at a later date, after both DWI and reference standard had been completed, then it was still important for a study to provide a description of whether the interpretation of each test was performed blind to the results of the other tests. Uninterpretable results produced in test accuracy studies are often not reported. Instead, they are simply removed from the analysis. This problem may lead to the biased assessment of the test characteristics. Whether bias will arise depends on the possible correlation between uninterpretable test results and the true disease status. If uninterpretable results occur randomly and are not related to the true disease status of the individual, these should not have any effect on test performance. However, whatever the cause of uninterpretable results, it is important that they are reported so that the impact of these results on test performance can be determined. Patients' withdrawal from the study can occur prior to the results of either or both of the index test and reference standard in diagnostic studies being available. If patients lost to follow-up differ systematically from those who remain in studies, then estimates of test performance may be biased. Therefore, it is important to report patients lost to follow-up. In brief, most studies in evaluating the diagnostic values of DWI for breast lesions had some clinical operational challenges. Therefore a systematic data reporting method such as the standards for reporting diagnostic accuracy (STARD) should be advocated to improve the quality of reporting test accuracy studies [48].

All of individuals showed that the mean ADC value of malignant lesions was lower than that of benign lesions. However, the reported mean ADC values of malignant and benign tumors ranged from 0.87 to 1.36 and 1.00 to 1.82 × 10^-3 ^mm^2^/s, respectively, resulting in recommended threshold values of ADC ranging from 0.90 to 1.76 × 10^-3 ^mm^2^/s. Even in the subgroup of studies using maximum b = 1000 s/mm^2^, the minimum and maximum threshold values were 1.10 × 10^-3 ^mm^2^/s [14,18] and 1.38 × 10^-3 ^mm^2^/s [43], respectively. The substantial variance of threshold values might be influenced by different b values, selection method, bias of patient selection, pathological characteristic of lesions and measurement of ADC values. This meta-analysis does not seem to predict or determine the unified threshold value to differentiate malignant and benign breast lesions because selection of the threshold value should be determined according to the purpose of examination. For example, a relatively higher threshold value may be recommended to minimize missing malignancy in breast cancer screening. If DWI is appended to the contrast-enhanced MRI, a relatively lower threshold value may be recommended to reduce false positive results.

## Conclusion

ADC measurement of DWI is useful for differentiation between malignant and benign breast lesions with pooled sensitivity of 0.84 and specificity of 0.84 in one homogenous subgroup of studies using maximum b = 1000 s/mm^2^, and the area under curve of sROC was 0.9085. DWI has a higher specificity but lower sensitivity compared to that of contrast-enhanced MRI. However, with all of methodological issues considered, results must be interpreted with caution. Large scale randomized control trials (RCTs) are necessary to assess and confirm its clinical value. A threshold value for malignant/benign lesions classification could not be made based on this study because it is influenced by different b values, bias of patient selection, lesions' pathological characteristics and ADC measurement. Selection of the threshold value should be determined according to the purpose of examination.

## Abbreviations

DWI: Diffusion-Weighted Imaging; sROC: Summary Receiver Operating Characteristic; ADC: Apparent Diffusion Coefficient; MRI: Magnetic Resonance Imaging; TP: True Positive; FP: False Positive; TN: True Negative; FN: False Negative; SEN: Sensitivity; SPE: Specificity; ACC: Accuracy; PPV: Positive Predictive Value; NPV: Negative Predictive Value; PLR: Positive Likelihood Radio; NLR: Negative Likelihood Ratio; *I*^2^: Inconsistency Index; NFS: Fail-Safe N; FEM: Fixed effect Mode; REM: Random Effect Mode; DOR: Diagnostic Odds Ratio; AUC: Area Under the Curve; 95%CI: 95% Confidence Interval; RCT: Randomized Control Trial.

## Competing interests

The authors declare that they have no competing interests.

## Authors' contributions

Chen X conceived of the study concept and participated in its design, quality assessment, statistical analysis, manuscript drafting and editing and approval for important intellectual concepts. Li WL and Bai ZL participated in the literature research, manuscript drafting and editing. Zhang YL participated in design and quality assessment. Wu Q participated in data acquisition and statistical analysis. Gou YM conceived of the study concept and participated in design, manuscript drafting and editing, data analysis and interpretation and approval for important intellectual concepts. All authors read and approved the final manuscript.

## Pre-publication history

The pre-publication history for this paper can be accessed here:

http://www.biomedcentral.com/1471-2407/10/693/prepub
